# Influence of Machining Parameters on the Surface Roughness and Tool Wear During Slot Milling of a Polyurethane Block

**DOI:** 10.3390/ma18010193

**Published:** 2025-01-05

**Authors:** Karolina Szadkowska, Norbert Kępczak, Wojciech Stachurski, Witold Pawłowski, Radosław Rosik, Grzegorz Bechciński, Małgorzata Sikora, Błażej Witkowski, Jakub Sikorski

**Affiliations:** Institute of Machine Tools and Production Engineering, Faculty of Mechanical Engineering, Lodz University of Technology, Stefanowskiego 1/15, 90-537 Lodz, Poland; szadkowska.kk@gmail.com (K.S.); wojciech.stachurski@p.lodz.pl (W.S.); witold.pawlowski@p.lodz.pl (W.P.); radoslaw.rosik@p.lodz.pl (R.R.); grzegorz.bechcinski@p.lodz.pl (G.B.); malgorzata.sikora@p.lodz.pl (M.S.); blazej.witkowski@p.lodz.pl (B.W.); jakub.sikorski@p.lodz.pl (J.S.)

**Keywords:** milling, polyurethane block, roughness *Ra* and *Rz*, wear of end mill *OE* and *VB*

## Abstract

The aim of the work was to investigate the influence of the machining parameters on the surface roughness and tool wear during slot milling of a polyurethane block (PUB). In the experiment, the influence of the cutting speed, the feed per tooth and the depth of cut on the roughness *Ra* and *Rz* of the milling slot surface and wear of the end mill was analyzed. A three-axis CNC milling machine Emco Concept Mill 55 was used to perform the study. After the machining, the values of parameters *Ra* and *Rz* were measured using the Hommel Tester T500 induction profilometer. Three polyurethane materials of different densities were considered: the Labelite 45, the Prolab 65 and the LAB 1000. The wear of the end mill was also examined for each of the tested materials by a workshop microscope. In conclusion, it was indicated how and to what extent the variation in the machining parameters affects the surface geometrical structure of a polyurethane plate. Moreover, the research results for the tested materials were compared with each other.

## 1. Introduction

Polyurethane (PU) materials are a group of plastics (polymers). They differ in chemical and physical properties depending on their composition and molecular weight. As a result, polyurethanes can have the form of cast resins, adhesives, varnishes, rubbers and foams and can be used in various applications, e.g., in biomedicine [1,2,3]. This paper concerns polyurethane foams.

Polyurethane foams of adequate density are characterized by low porosity, good machinability and good dimensional stability, thanks to which they are widely used in prototyping as a less expensive alternative to metallic materials. Polyurethane foams are used, for example, in model-making and architecture to make mock-ups or reference models [4,5]. Another well-known use is the manufacture of testing instruments for automotive parts [6,7].

The above-mentioned applications of polyurethane foams require them to be machined using CNC machine tools [8] or industrial robots [7] supported by CAM software [8]. The most common form of the semi-finished part is a polyurethane block (PUB), and the final shape of the workpiece is obtained by milling with various types of cutting tools: face mills, end mills or ball end mills, depending on the shape of the surface to be machined [9,10,11,12]. At the same time, one of the important aspects of the PUB machining process is the quality of the machined surfaces, on the one hand, and the durability of the cutting tools used, on the other hand.

Surface roughness is one of the most important ingredients for assessing the quality of a part. Measurement of *Ra* or *Rz* parameter is a method commonly used to describe surface roughness. This method can be applied to every solid material: metals, non-metals, plastics, composites, etc. The paper of Mitalova et al. [13] presented a surface topography evaluation of wood plastic composite (WPC) materials after turning. Zhang et al. [14] in their paper investigated the surface roughness prediction and roughness reliability evaluation of CNC milling based on surface topography simulation. To verify the accuracy of the surface topography and surface roughness model, experimental milling tests of an aluminum alloy 7050 were conducted. The paper of Duplak et al. [15] investigated surface roughness and microhardness of C45 steel material using high-speed machining (HSM).

It should be noted that, during the milling of metallic materials, the machining conditions, including, among other things, the cutting parameters and the properties of the workpiece material, play a decisive role in PUB machining. Their relevance is indicated by the few available studies.

The paper of Catherine et al. [6] investigated the effect of machining conditions on the surface roughness of a polyurethane block after milling using two- and four-flute high-speed steel (HSS) end mills. The tests were carried out without the use of a coolant because it had been shown that the coolant had been absorbed by the porous material, which, over time, had caused geometric distortions in the surface and a deterioration in surface roughness. It was observed that lower surface roughness could be obtained by dry milling (without coolants). Under these conditions, machining was carried out by varying the values of cutting parameters such as depth of cut (back engagement), feed rate, width of cut (working engagement), spindle rotational speed and vertical cutting speed. Experience has shown that roughness was most influenced by changes in the width of cut. The smallest roughness was achieved for the lower width of cut, and the higher value of the parameter resulted in the highest roughness value out of all the studied machining parameters. Subsequently, the following parameters had a decreasing effect on the roughness of the resulting surface: cutting speed, feed rate, depth of cut and spindle rotational speed.

The paper of Hafner et al. [16] investigated the effect of milling process parameters for polyurethane foam of different densities on its surface roughness. Dry machining was carried out using a two-flute end mill, milling the surfaces of the samples using various values of feed per tooth, spindle rotational speed and depth of cut. The study showed that, of all the parameters determining the machining conditions, the density of the workpiece material had the greatest influence on surface roughness. It was observed that the higher the density of the polyurethane foam, the lower the surface roughness (*Ra*) after milling. It was also shown that another important parameter was the spindle rotational speed, an increase of which resulted in a decrease in surface roughness. Equally important was feed per tooth, which was changed during the later stages of the experiment when milling two foams of different densities at constant and maximum spindle rotational speed. Based on experimental results, it was found that increasing the feed per tooth increased the roughness of the machined surface, i.e., reduced its quality.

Utomo et al., in their paper [8], identified the cutting parameters as having a significant influence on the surface roughness (*Rz*) and the flank wear (*VB*) obtained after milling. These parameters included spindle rotational speed and the resulting cutting speed, depth of cut and feed rate. At the same time, the authors of the paper [8] stated that due to the different properties of polyurethane foams, milling required a careful selection of the cutting parameters in each case in order to achieve the required quality of the machined surface. In this aspect, it is also important to use tools with sharp cutting edges and the smallest possible rounding radius.

In addition, the authors of the paper [8] pointed out that the correct choice of machining conditions during PUB milling was indicated by the formation of a continuous chip, resulting in a better quality of the machined surface. In contrast, obtaining intermittent chips is associated with surface damage in the form of porous holes that result from crack propagation rather than actual cutting. It is worth noting that the formation of different types of chips and the final quality of the machined surface are influenced by the depth of cut and the cell size of the machined polyurethane foam. Machining with appropriate parameters can reduce surface roughness and ensure continuous chip formation at a depth of one to two widths of the PU cell.

A significant effect of the chips produced during PU milling on tool life was pointed out by Muflikhun et al. in their paper [17]. Machining was carried out using end mills made of HSS with a constant spindle rotational speed of 3000 rpm and a variable feed rate: 150, 300, 450, 600 mm/min and depth of cut: 0.5, 1, 1.5, 2 mm. The authors showed that, for each feed rate, increasing the depth of cut resulted in more chips adhering to the cutting edges. This shortens tool life and reduces the quality of the machined surface.

In summary of the above, it should be concluded that the surface roughness of the polyurethane foam obtained by milling is most influenced by its density and the machining conditions. The parameters of the latter, especially the cutting parameters, are selected individually, depending on the properties of the machined material. Most of the researchers cited above used one roughness parameter—*Ra* or *Rz*—to assess surface quality. However, using more than one parameter, each determined differently based on the roughness profile, enables a better assessment of the quality of the machined surface. This is confirmed by other research, e.g., [18,19]. In addition, milling cutting tools made of HSS with two to four cutting blades are most commonly used for PUB milling, which, due to the large chip grooves, enables efficient run-off of a large volume of chips and slower clogging of the spaces between the cutting blades. It should be noted that only a few studies have addressed the subject of tool wear as a function of the machining conditions. They limit themselves to a general statement about the presence of wear, without providing specific wear indicators. It seems that, because of the possibility of different forms of wear [20,21], it is appropriate to measure the wear marks on the flank face and tooth face of the blades, as presented in other studies, e.g., [22,23,24].

Taking this into account, the aim of the research described in this paper was to investigate the effects of polyurethane foam density and cutting parameters on the roughness of the obtained surface and the wear of cutting tool edges. During the tests, three PU blocks differing in density were dry-milled using the same sets of variable cutting parameters each time, comprising four cutting speeds *v_c_*, six values of feed per tooth *f_z_* and five depths of cut *a_p_*. Two-flute end mills made of HSS, with a standard geometry, were used as the cutting tool. Each PUB was machined with a new cutting tool. Two roughness parameters, *Ra* and *Rz*, were used to assess surface roughness. The tool wear was determined by measuring the flank wear (*VB*) and outside edge wear (*OE*), respectively. Taking into account surface quality, the final ranges of cutting parameters were determined. The final section of the paper summarizes the results of the tests and presents the final conclusions.

## 2. Materials and Methods

### 2.1. Materials

**Figure 1:**
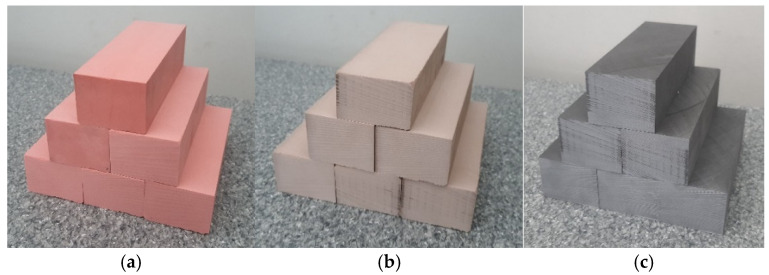
The view of samples: (**a**) Labelite 45 PK, (**b**) Prolab 65 and (**c**) LAB 1000.

Three polyurethane materials were used for the tests: Labelite 45 PK, Prolab 65 and LAB 1000. They are characterized by good machinability and dimensional stability. Their physical and mechanical properties are shown in Table 1 based on [25,26,27]. Figure 1 shows the cuboidal material samples prepared for testing, measuring 135 × 40 × 50 mm.

### 2.2. Machine Tool

**Figure 2:**
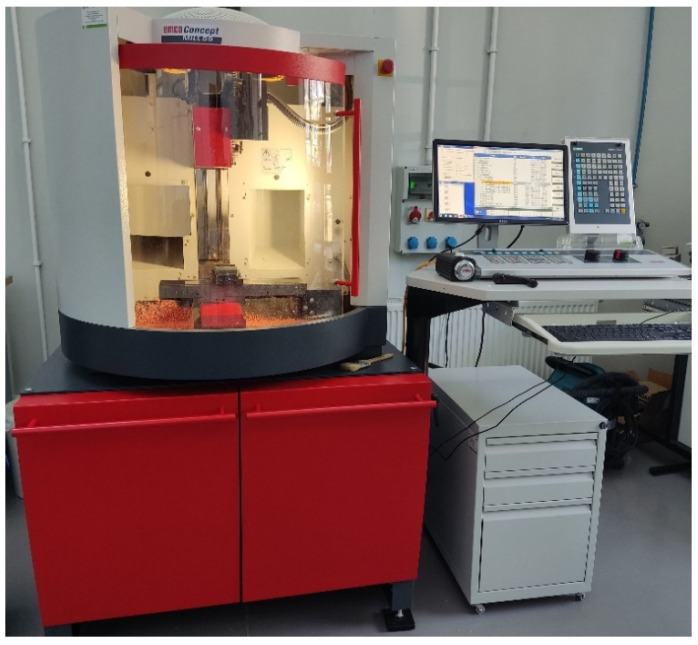
The view of EMCO Concept Mill 55.

The machine tool used for the tests was a three-axis CNC milling machine from EMCO with the designation Concept Mill 55. Table 2 presents the basic machine data (based on [28]), while Figure 2 shows a view of the machine.

### 2.3. Cutting Tool

**Figure 3:**
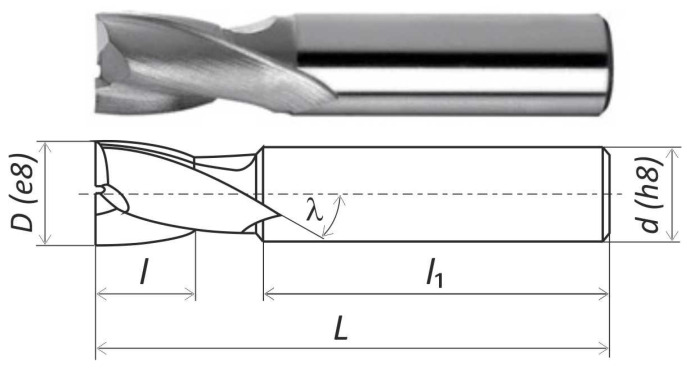
End mill 16 HSS DIN 327 [29].

The grooves were milled using double-blade end milling shank cutting tools with a diameter of 16 mm and the designation NFPg (according to DIN 327) made of HSS without wear coating. Based on [29], the technical specifications of the cutting tools are listed in Table 3, and their illustrations are shown in Figure 3. A new cutting tool was used for each tested material so that the wear of its blades could be assessed later in the study.

### 2.4. Cutting Conditions

**Figure 4:**
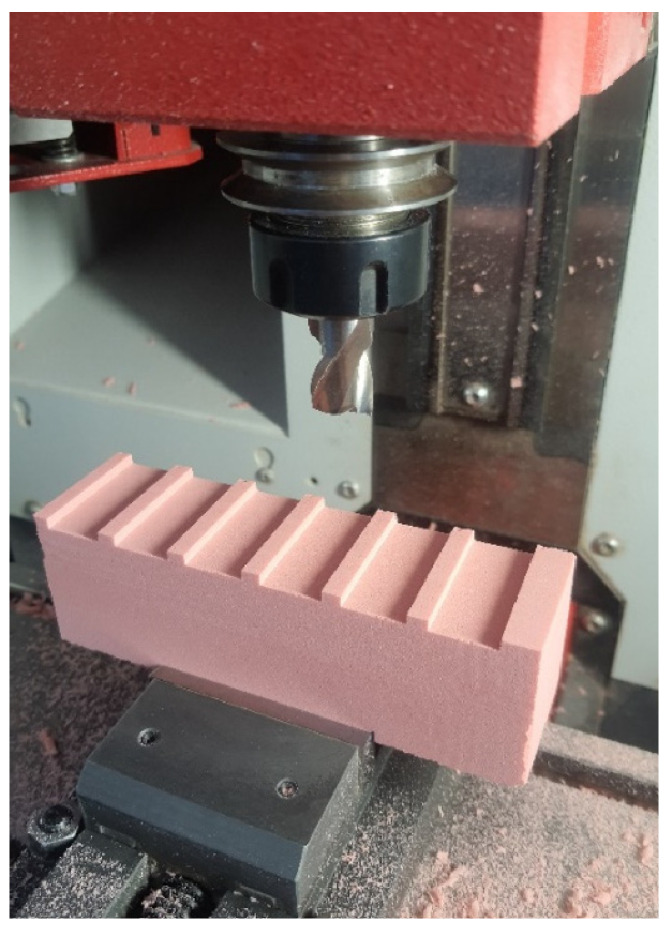
The view of a milled sample.

The study began with the milling process. For this purpose, samples of the selected material were clamped in a precision machine vice and placed on the machine table as shown in Figure 4. The samples were set in the middle of a vice to minimize deflection on both sides of the sample. Moreover, both chucks of vice were equipped with locks to protect against the displacement of a sample. The grooves were then milled using 120 sets of variable cutting parameters; the values of which are shown in Table 4. The range of cutting parameters was selected based on the limitations of the machine tool. The maximum rotational speed of a spindle is 3500 rpm, and the maximum feed rate is 2000 mm/min. To avoid exceeding the limits, the diameter of the tool was selected to be 16 mm. This is also the maximum possible size of an end mill, which can be used on this machine tool. As evidenced by Figure 4, the size of a single sample was sufficient to make six grooves. The milling process was carried out without coolants.

After a whole series of milling operations (120 grooves) on samples of a given material, the surface roughness of the groove bottom and cutting tool wear was measured.

The procedure described above was repeated using samples made of the subsequent material.

### 2.5. Surface Roughness Measurements

Measurements of groove bottom roughness after milling were carried out using a Hommel Tester T500 (Hommelwerke GmbH, Schwenningen, Germany) portable contact profilometer with a T5E stylus [30]. The measuring conditions were chosen in accordance with EN ISO 4288 and are summarized in Table 5.

**Figure 5:**
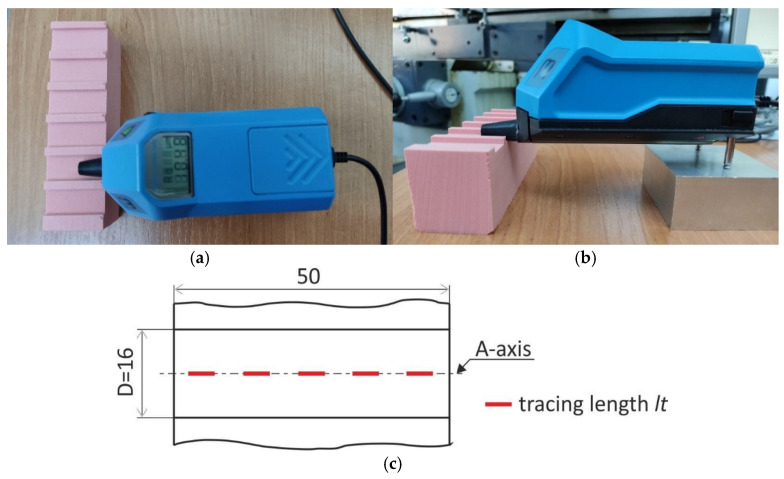
The view of Hommel Tester T500: (**a**) upper view, (**b**) side view and (**c**) location of tracing length on the groove axis.

The relative positioning of the sample and profilometer during the measurement is shown in Figure 5a,b. The stylus moved along the groove bottom surface along five measuring sections *l_t_* distributed along the *A*-axis at the locations indicated in Figure 5c. In the considered case, the *A*-axis is identical to the line of travel of the axis of rotation of the cutting tool during milling, so its direction is consistent with the direction of the feed motion of the cutting tool.

The above measurement of the groove bottom enabled the determination of surface roughness parameters that could be used to evaluate roughness. Two roughness parameters were used in accordance with PN-ISO 4287—the amplitude parameter *Ra* and the vertical parameter *Rz*. Since, as mentioned earlier, each surface was measured five times, the arithmetic average of the five measurements was taken as the *Ra* and *Rz* values representative of the individual surface.

### 2.6. Wear Measurement

**Figure 6:**
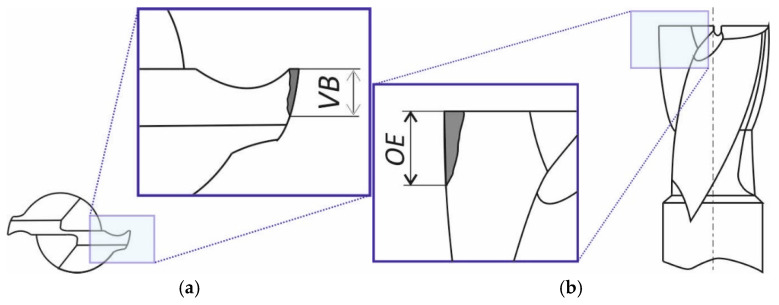
Wear measurements on end mill cutting tool blades: (**a**) flank wear *VB* and (**b**) outside edge wear *OE*.

A visual inspection of the cutting tool blades showed signs of wear in the form of abrasion on the flank face and rake face. For this reason, it was decided to take measurements only in these areas and determine two wear indices, *VB* on the flank face and *OE* on the rake face, respectively. These indices were adopted from descriptions of studies in the literature [23,31]. Their location on cutting tool surfaces is shown in Figure 6.

## 3. Results and Discussion

### 3.1. Surface Roughness

**Figure 7:**
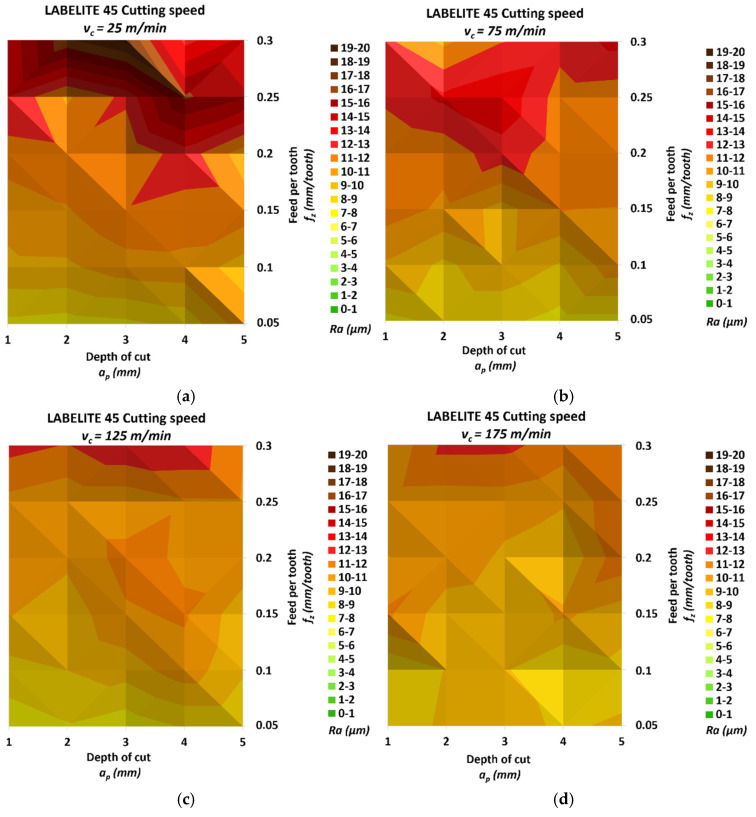
Results of surface roughness *Ra* for Labelite 45 PK: (**a**) *v_c_* = 25 m/min, (**b**) *v_c_* = 75 m/min, (**c**) *v_c_* = 125 m/min and (**d**) *v_c_* = 175 m/min.

Figure 7 shows the values of the *Ra* parameter obtained by measuring the surface roughness of samples made of Labelite 45 PK.

The smallest *Ra* was 7.34 μm, and it was measured for a surface milled with a cutting speed *v_c_* = 125 m/min, the smallest feed per tooth *f_z_* = 0.05 mm/tooth and the smallest depth of cut *a_p_* = 1 mm. The highest *Ra* was 18.29 μm, and it was achieved after milling with the smallest cutting speed *v_c_* = 25 m/min, the highest feed per tooth *f_z_* = 0.3 mm/tooth and the depth of cut *a_p_* = 3 mm. The relative increase between the above *Ra* values was 149.18%. The relative increases in roughness for the individual cutting speed groups in the direction of increasing speed, in turn, were 141.83%, 80.73%, 76.11% and 53.45%, respectively. The change in *Ra* due to the change in feed per tooth for the different cutting speed groups, starting from the lowest, was 135.90%, 74.44%, 75.95% and 34.95%, respectively. Similarly, for depth of cut, the change reached values of 38.77%, 16.55%, 22.70% and 23.33%. The steepest increase in *Ra* was observed for a cutting speed of *v_c_* = 25 m/min and for a feed per tooth greater than *f_z_* = 0.25 mm/tooth.

**Figure 8:**
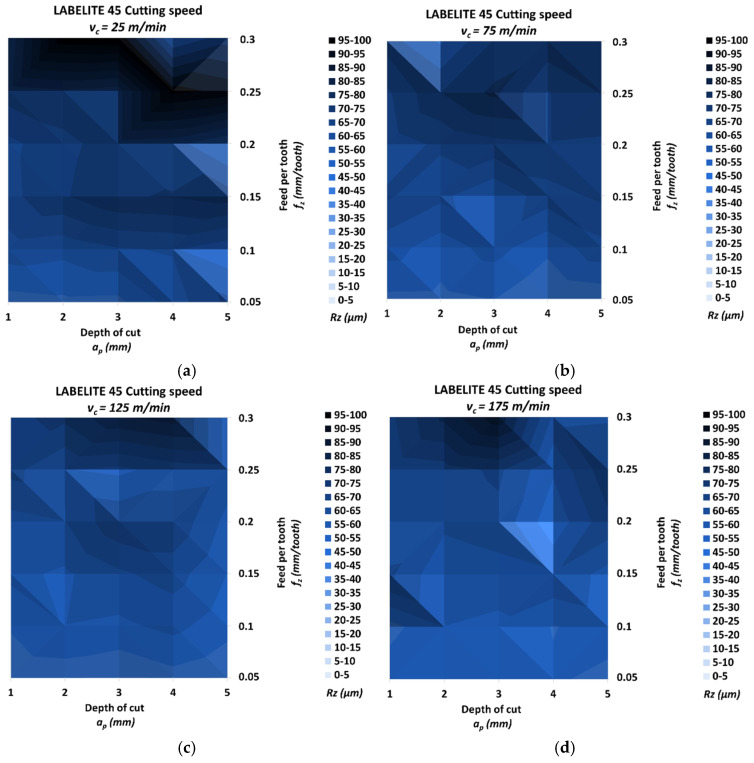
Results of surface roughness *Rz* for Labelite 45 PK: (**a**) *v_c_* = 25 m/min, (**b**) *v_c_* = 75 m/min, (**c**) *v_c_* = 125 m/min and (**d**) *v_c_* = 175 m/min.

Figure 8 shows the values of the roughness parameter Rz obtained for the milled surface of samples made of Labelite 45 PK.

The lowest *Rz* was 40.12 μm, and it was achieved for the cutting speed *v_c_* = 75 m/min, the lowest feed per tooth *f_z_* = 0.05 mm/tooth and the depth of cut *a_p_* = 4 mm. The highest *Rz* was 99.17 μm, and it was achieved for the lowest cutting speed *v_c_* = 25 m/min, the highest feed per tooth *f_z_* = 0.3 mm/tooth and the depth of cut *a_p_* = 3 mm. The relative increase for these values was 147.17%. The *Rz* increment for cutting speeds *v_c_* = 25/75/125/175 m/min was 131.44%, 89.80%, 91.87% and 93.80%, respectively. The relative spread of the parameter *Rz* for the different cutting speed groups due to the change in feed per tooth was, in the direction of increasing cutting speed, 127.55%, 83.84%, 84.17% and 80.54%, respectively. Similarly, the relative spread due to the change in depth of cut was 52.22%, 29.04%, 20.90% and 42.31%. Figure 8a,d show a rapid increase in roughness for feed per tooth greater than *f_z_* = 0.25 mm/tooth. For the lowest feed per tooth *f_z_* = 0.05 mm/tooth, the lowest values of the parameter *Rz* were recorded, with the exception of the values for the lowest cutting speed *v_c_* = 25 m/min and depth of cut *a_p_* = 4 mm and *a_p_* = 5 mm.

**Figure 9:**
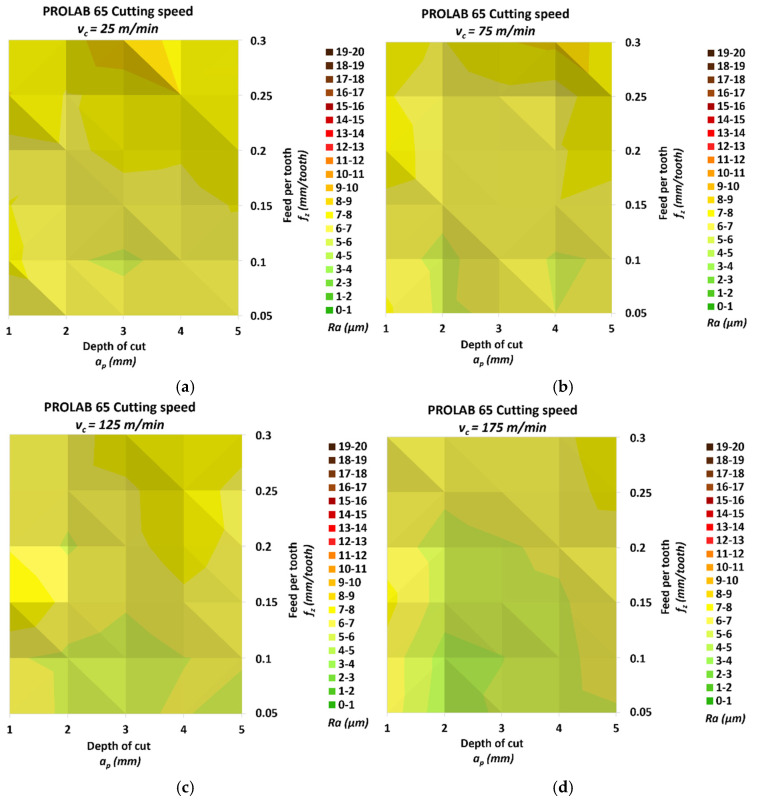
Results of surface roughness *Ra* for Prolab 65: (**a**) *v_c_* = 25 m/min, (**b**) *v_c_* = 75 m/min, (**c**) *v_c_* = 125 m/min and (**d**) *v_c_* = 175 m/min.

Figure 9 shows the results of measuring the roughness *Ra* of the milled surface for the Prolab 65 material.

The smallest *Ra* was 4.28 μm, and it was measured for the smallest cutting speed *v_c_* = 175 m/min, the smallest feed per tooth *f_z_* = 0.05 mm/tooth and the depth of cut *a_p_* = 2 mm. The highest *Ra* was 8.98 μm, and it was achieved for the lowest cutting speed *v_c_* = 25 m/min, the highest feed per tooth *f_z_* = 0.3 mm/tooth and the depth of cut *a_p_* = 3 mm. The relative increase between these values was 110.05%. The relative increases in roughness for the individual cutting speed groups in the direction of increasing speed were 75.53%, 47.96%, 45.72% and 54.62%, respectively. The change in *Ra* due to the change in feed per tooth for the different speed groups, starting from the lowest, was 45.60%, 37.40%, 37.22% and 38.85%, respectively. Similarly, for the depth cut, the change reached values of 35.50%, 23.13%, 20.07% and 20.15%. Figure 8d shows that for the depth of cut *a_p_* = 2 mm and *a_p_* = 3 mm and a feed per tooth lower than or equal to *f_z_* = 0.2 mm/tooth, the roughness values are significantly lower than for the other depths of cut.

**Figure 10:**
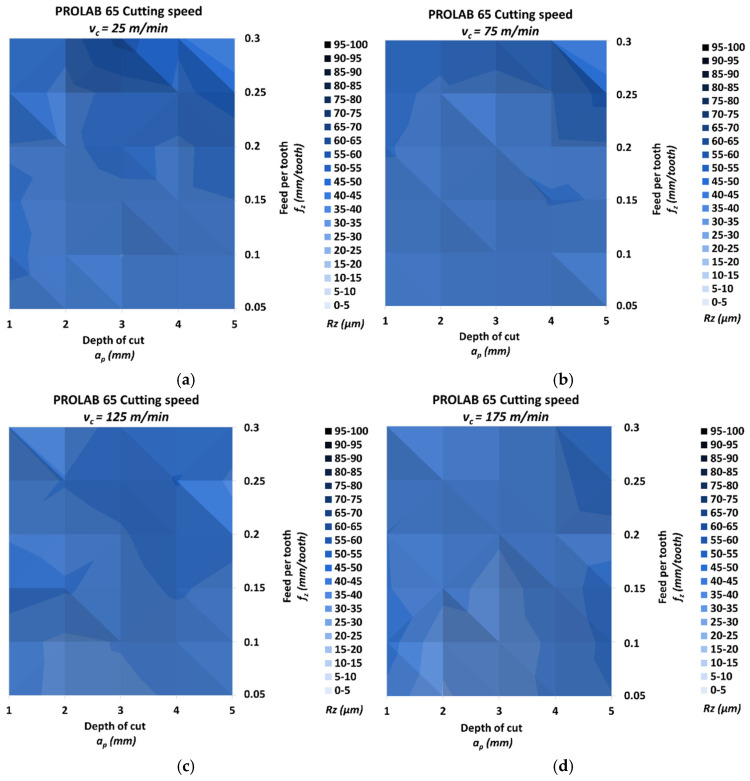
Results of surface roughness *Rz* for Prolab 65: (**a**) *v_c_* = 25 m/min, (**b**) *v_c_* = 75 m/min, (**c**) *v_c_* = 125 m/min and (**d**) *v_c_* = 175 m/min.

Figure 10 shows the results of measuring the roughness *Ra* of the milled surface for the Prolab 65 material.

The lowest *Rz* was 29.62 μm, and it was achieved for the highest cutting speed *v_c_* = 175 m/min, the lowest feed per tooth *f_z_* = 0.05 mm/tooth and the depth of cut *a_p_* = 2 mm. The highest *Rz* was 52.02 μm, and it was achieved for the lowest cutting speed *v_c_* = 25 m/min, the highest feed per tooth *f_z_* = 0.3 mm/tooth and the depth of cut *a_p_* = 3 mm. The relative increase for these values was 75.59%. The *Rz* increase for cutting speeds *v_c_* = 25/75/125/175 m/min was 49.45%, 34.59%, 32.19% and 49.13%, respectively. The relative spread of the parameter *Rz* for the different cutting speed groups due to the change in feed per tooth was, in the direction of increasing speed, 34.49%, 27.78%, 23.17% and 38.24%, respectively. Similarly, the relative spread due to the change in the depth of cut was 27.66%, 22.79%, 14.96% and 20.16%. The highest and lowest *Rz* were obtained for the same machining conditions as for Ra. Similarly, for the cutting speed *v_c_* = 175 m/min, depth of cut *a_p_* = 2 mm and *a_p_* = 3 mm for a feed per tooth lower than or equal to *f_z_* = 0.2 mm/tooth, the *Rz* is significantly lower than for the other depths of cut.

**Figure 11:**
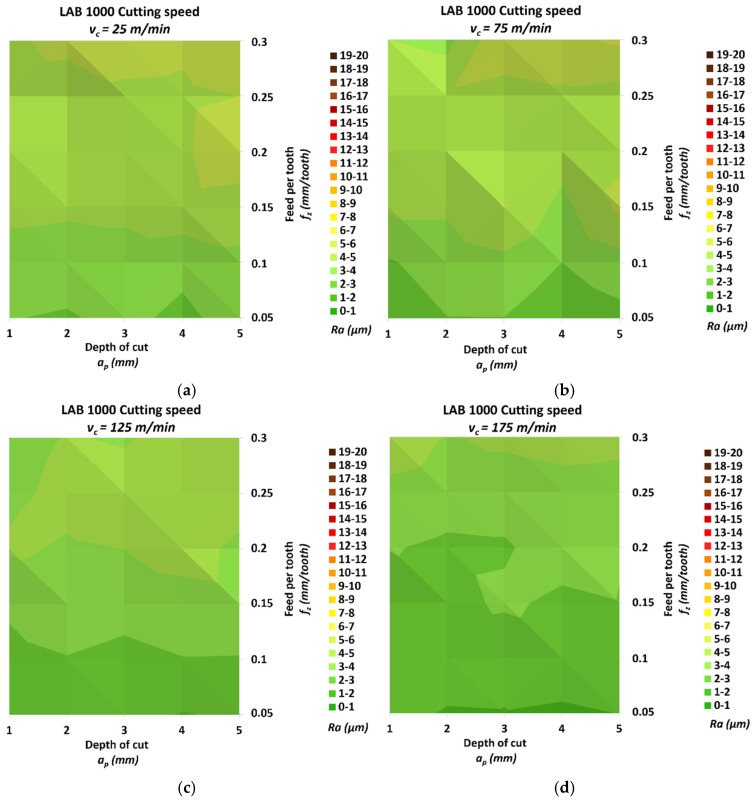
Results of roughness *Ra* for LAB 1000: (**a**) *v_c_* = 25 m/min, (**b**) *v_c_* = 75 m/min, (**c**) *v_c_* = 125 m/min and (**d**) *v_c_* = 175 m/min.

Figure 11 shows the results of measuring the roughness Ra of the milled surface for the LAB 1000 material.

The smallest *Ra* was 1.84 μm, and it was measured for the smallest cutting speed *v_c_* = 175 m/min, the smallest feed per tooth *f_z_* = 0.05 mm/tooth and the depth of cut *a_p_* = 4 mm. The highest *Ra* was 6.14 μm, and it was achieved for the cutting speed *v_c_* = 75 m/min, the highest feed per tooth *f_z_* = 0.3 mm/tooth and the depth of cut *a_p_* = 5 mm. The relative increase between these values was 233.27%. The relative increases in roughness for the individual speed groups in the direction of increasing speed were 154.97%, 153.23%, 168.66% and 113.05%, respectively. The change in *Ra* due to the change in feed per tooth for the different speed groups, starting from the lowest, was 137.07%, 137.48%, 153.64% and 90.49%, respectively. Similarly, for the depth of cut, the change reached values of 37.50%, 41.94%, 53.71% and 24.43%. It can be seen from the figure above that, for the highest cutting speed *v_c_* = 175 m/min, the achieved roughness values were mostly lower than those obtained for the other speeds.

**Figure 12:**
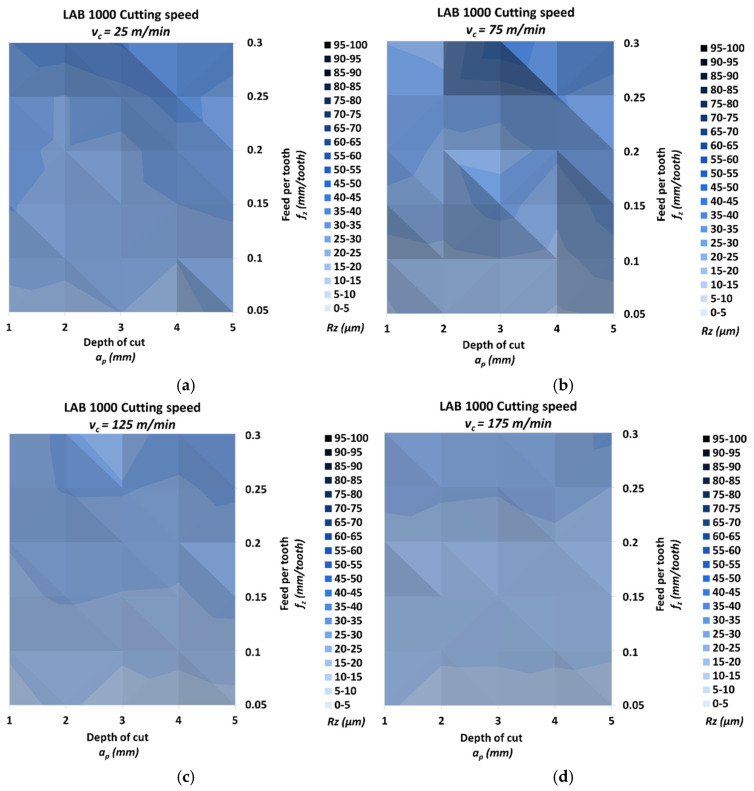
Results of surface roughness *Rz* for LAB 1000: (**a**) *v_c_* = 25 m/min, (**b**) *v_c_* = 75 m/min, (**c**) *v_c_* = 125 m/min and (**d**) *v_c_* = 175 m/min.

Figure 12 shows the results of measuring the roughness *Rz* of the milled surface for the LAB 1000 material.

The lowest *Rz* was 11.51 μm, and it was achieved for the highest cutting speed *v_c_* = 175 m/min, the lowest feed per tooth *f_z_* = 0.05 mm/tooth and the depth of cut *a_p_* = 4 mm. The highest *Rz* was 39.20 μm, and it was achieved for the cutting speed *v_c_* = 75 m/min, the highest feed per tooth *f_z_* = 0.3 mm/tooth and the depth of cut *a_p_* = 3 mm. The relative increase for these values was 240.54%. The *Rz* increment for cutting speeds *v_c_* = 25/75/125/175 m/min was 130.29%, 147.68%, 173.59% and 117.11%, respectively. The relative spread of the parameter *Rz* for the different cutting speed groups due to the change in feed per tooth was, in the direction of increasing cutting speed, 95.27%, 138.90%, 125.87% and 116.32%, respectively. Similarly, the relative spread due to the change in depth of cut was 27.17%, 57.73%, 26.21% and 25.27%.

The results of surface roughness measurements after milling discussed above indicate that the main cutting parameters that affect the value of the surface roughness parameters *Ra* and *Rz* are the cutting speed *v_c_* and the feed per tooth *f_z_*. As *v_c_* increases, the values of the *Ra* and *Rz* parameters decrease, while as *f_z_* increases the values of the *Ra* and *Rz* parameters also increase. At the same time, it is worth noting that changing the depth of cut *a_p_* has no significant effect on the results.

Furthermore, it should be noted that the density of the milled material has the greatest impact on surface roughness parameters *Ra* and *Rz*. For the material with the lowest density (Labelite 45 PK), the values of *Ra* and *Rz* were the highest. In contrast, the material with the highest density (LAB 1000) had the lowest values for both surface microgeometry parameters. This can be explained by the fact that the lower the density of the machined material, the more porous it is, and therefore, its natural roughness is greater.

### 3.2. Comparison of Ra and Rz Values

**Figure 13:**
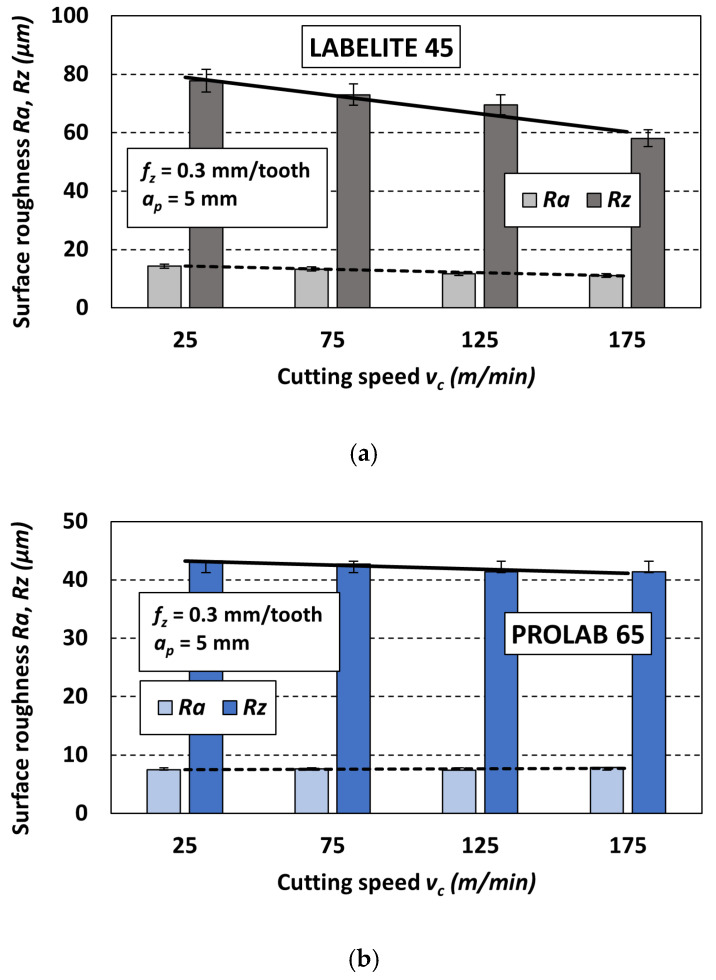

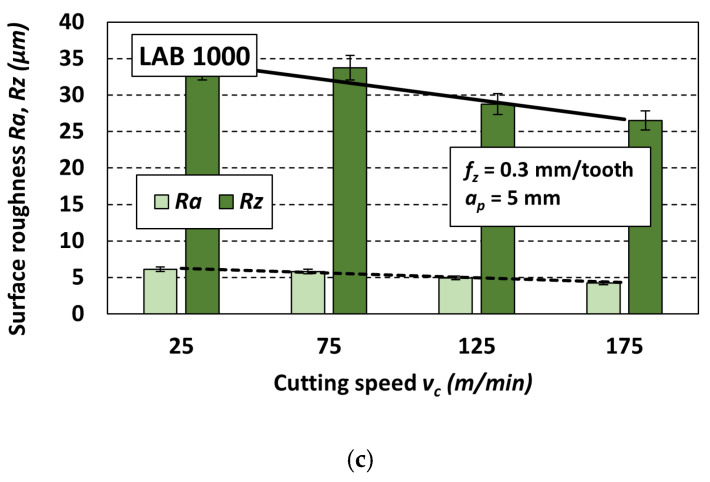
Example values of the roughness parameters *Ra* and *Rz* as a function of the cutting speed *v_c_* for different PUB materials: (**a**) Labelite 45 PK, (**b**) Prolab 65 and (**c**) LAB 1000; values of constant cutting parameters *f_z_* = 0.3 mm/tooth, *a_p_* = 5 mm.

To better visualize the changes in the obtained surface roughness parameters (*Ra* and *Rz*), bar charts were created. An example set is shown in Figure 13. The set includes three charts, each one corresponding to a different PUB material: Labelite 45 PK (Figure 13a), Prolab 65 (Figure 13b) and LAB 1000 (Figure 13c), which have been milled with the same constant cutting parameters: *f_z_* = 0.3 mm/tooth and *a_p_* = 5 mm, and four different cutting speeds *v_c_*. It should be noted that the trend of changes in the *R* parameter values was similar for changes in cutting speed for other sets of constant cutting parameters.

As can be seen in Figure 13, the trend line has a significantly steeper slope for the *Rz* parameter compared to the *Ra* parameter. This is due to the greater differences between the *Rz* parameter values obtained for different cutting speeds. The reason for this can be attributed to the different methods of determining the values of each parameter. The *Rz* parameter is defined as the sum of the height of the highest peak and the depth of the lowest valley within the sampling length, while the *Ra* parameter is the arithmetic mean of the profile ordinates. Therefore, the *Rz* parameter is more sensitive to the peaks and valleys of the measured roughness profile. This also explains its several times higher value compared to the *Ra* parameter obtained for the same set of cutting parameters.

Based on the charts presented in Figure 13, it should be noted that changing the cutting speed *v_c_* causes changes in the values of the obtained surface roughness parameters. This observation is significant because, from a theoretical point of view, changing the cutting speed should not affect the surface roughness. It should only result from the replication of the cutting tool geometry and feed rate [20]. However, in the considered case, this effect is visible and may be related to the density of the machined PUB material. For the material with the lowest density (Labelite 45 PK), increasing the cutting speed clearly reduces the values of both roughness parameters (Figure 13a). The same trend was observed for the material with the highest density (LAB 1000); increasing *v_c_* clearly reduces the values of the *Ra* and *Rz* parameters (Figure 13c). For the third material with medium density (Prolab 65), increasing the cutting speed does not significantly affect the change in both surface roughness parameters, resulting in a trend line with a slight slope (Figure 13b).

The above observations were confirmed by conducting significance tests on the effect of cutting speed during milling, depending on the applied PUB material. The tests were carried out using a Complete Random Design (CRD) statistical program [32]. According to standard practice, the null hypothesis was assumed, stating no effect of the analyzed input factor on the output factor. Then, the calculated value was determined according to the Fisher–Snedecor F-statistic and compared with the critical value *F_kr_* listed in the tables (significance level *α* = 0.05). The comparison showed that within the accepted range of variability, cutting speed significantly affects the *Ra* and *Rz* parameter values during the machining of Labelite 45 PK and LAB 1000 materials (*F* ≥ *F_kr_*) and does not significantly affect the milling of Prolab 65 material (*F* ≤ *F_kr_*).

As mentioned earlier, the described effect of cutting speed on the *Ra* and *Rz* parameter values depending on the type of PUB material can be linked to its density. Generally, the literature contains numerous studies indicating the dependence of the obtained *R* parameters on changes in cutting speed [33,34]. Interestingly, depending on the adopted research conditions, increasing the cutting speed can lead to both an increase and a decrease in surface roughness [8,35,36]. The authors of these studies explain this by various phenomena occurring at the interface between the cutting edge and the machined material, depending on the properties of the last one. Therefore, a probable reason why the surface roughness decreases with increasing cutting speed during the milling of the material with the lowest density (Labelite 45 PK) is the smaller volume of removed material, which translates into fewer chips and a more efficient process of their removal from the tool’s chip grooves and the cutting zone.

**Figure 14:**
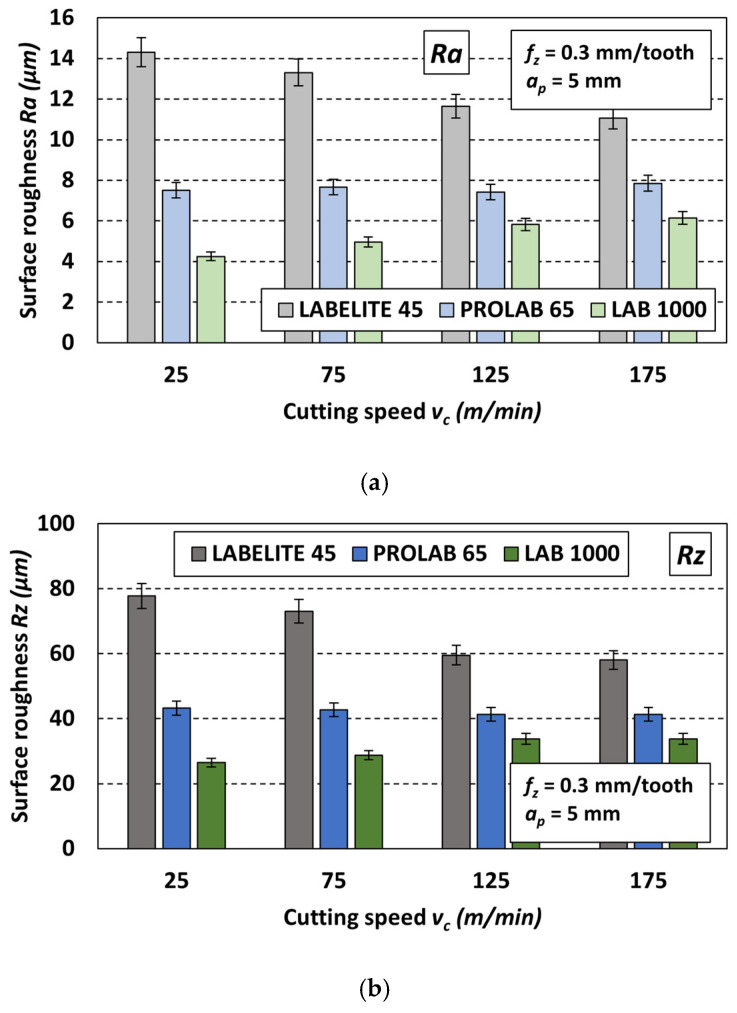
Influence of type of PUB material and variable cutting speed *v_c_* on the surface roughness: (**a**) *Ra* and (**b**) *Rz*; values of constant cutting parameters *f_z_* = 0.3 mm/tooth, *a_p_* = 5 mm.

The effect of the density of the material from which the PUB is made on surface roughness is clearly visible when comparing the *R* values obtained using the same set of cutting parameters. Figure 14 shows example charts of changes in the *Ra* parameter (Figure 14a) and *Rz* parameter (Figure 14b) after milling with four cutting speeds *v_c_* and with constant cutting parameters: *f_z_* = 0.3 mm/tooth and *a_p_* = 5 mm. It should be noted that the nature of changes in the *R* parameter values under the influence of changes in cutting speed for other sets of constant cutting parameters was similar.

As shown in the charts in Figure 14, for each set of parameters including a constant *f_z_* and *a_p_* value and one of the four *v_c_* values, the highest roughness parameter *R* was obtained after milling the PUB with the lowest density (Labelite 45 PK), and the lowest for the surface of the PUB with the highest density (LAB 1000). This is likely due to the fact that the lower the material density, the greater the proportion of depressions on the measured surface compared to the surface of the higher-density material. The replication of this shape by the profilometer’s measuring tip stylus results in higher surface roughness parameters. Increasing the material density effectively smooths the measured surface and reduces its roughness.

Further analysis of the results was conducted for cases where the variable parameter was the depth of cut *a_p_*. Similarly to the previous analysis, significance tests were performed on the effect of the variable cutting parameter depending on the applied PUB material, using a Complete Random Design statistical program and the Fisher–Snedecor F-statistic (significance level *α* = 0.05). The comparison showed that within the accepted range of variability, the depth of cut does not significantly affect the milling of any of the three materials (*F* ≤ *F_kr_*). Therefore, the authors decided not to show the comparison charts.

**Figure 15:**
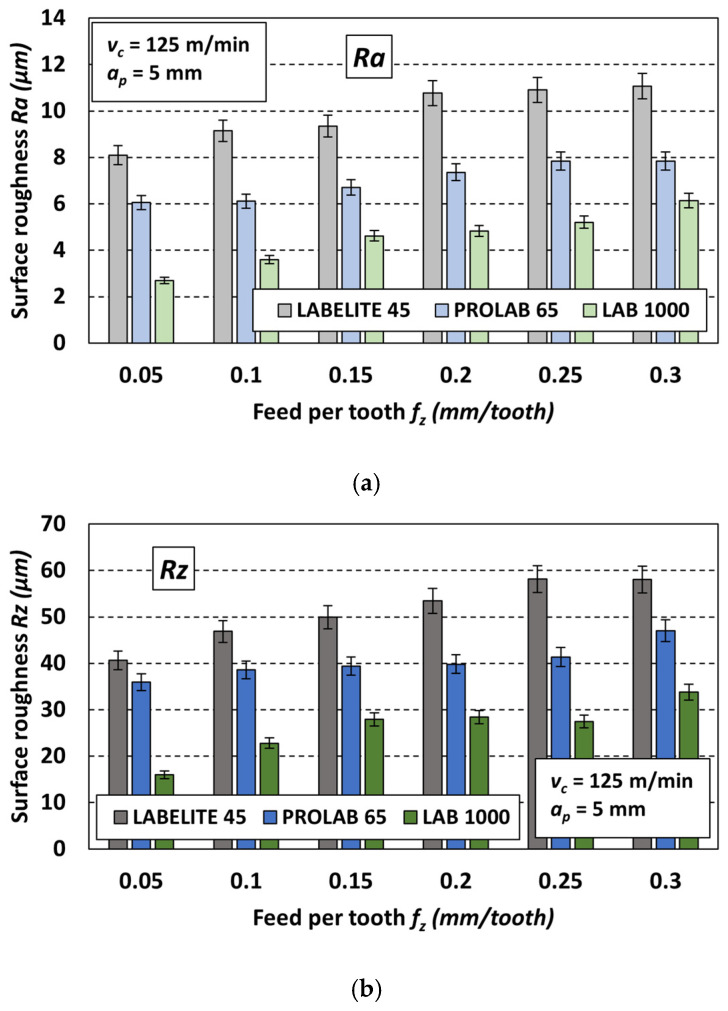
Influence of type of PUB material and variable depth of cut *a_p_* on the surface roughness: (**a**) *Ra* and (**b**) *Rz*; values of constant cutting parameters *f_z_* = 0.3 mm/tooth, *v_c_* = 125 m/min.

Similarly to the previously discussed case (variable *v_c_*), significant differences were observed between the values of both parameters obtained during the milling of the three materials with the same sets of cutting parameters. Figure 15 shows example charts of changes in the *Ra* parameter (Figure 15a) and *Rz* parameter (Figure 15b) after milling with five depths of cut *a_p_* and with constant cutting parameters: *f_z_* = 0.3 mm/tooth and *v_c_* = 125 m/min. It should be noted that the nature of changes in the *R* parameter values under the influence of changes in the depth of cut for other sets of constant cutting parameters was similar.

As shown in the charts in Figure 15, for each set of parameters including a constant *f_z_* and *v_c_* value and one of the five *a_p_* values, the highest roughness parameter value was obtained after milling the PUB with the lowest density (Labelite 45 PK), and the lowest for the surface of the PUB with the highest density (LAB 1000). Similar to the previously discussed case, this observed property can be explained by the material structure, which, in the case of the lower-density material, has a greater number of depressions on the measured surface. Increasing the material density smooths the measured surface and reduces its roughness.

**Figure 16:**
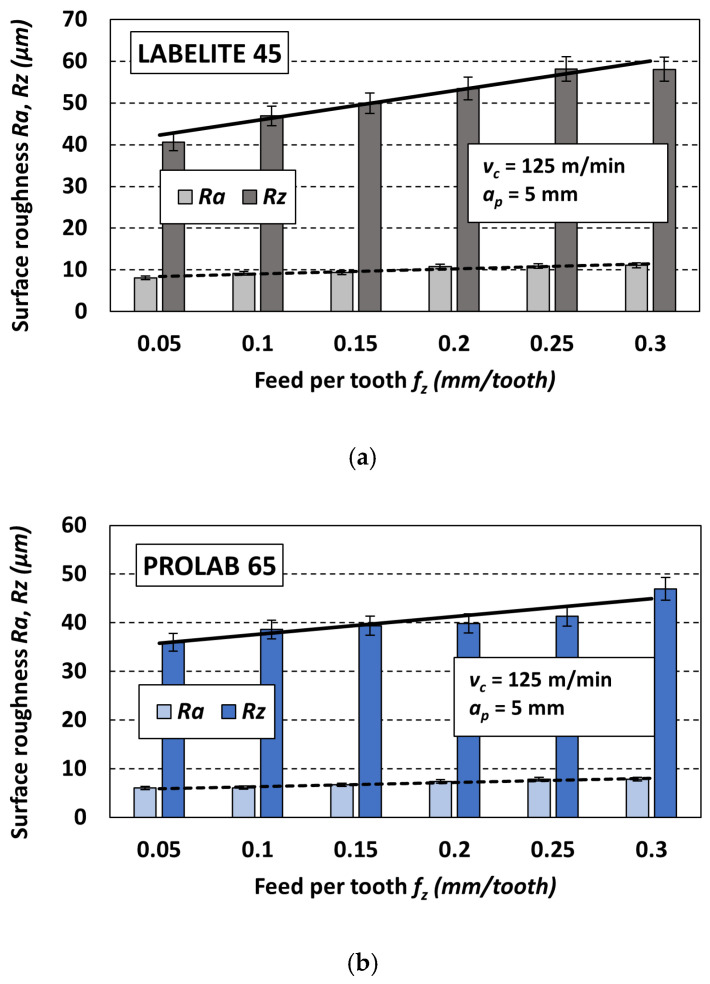

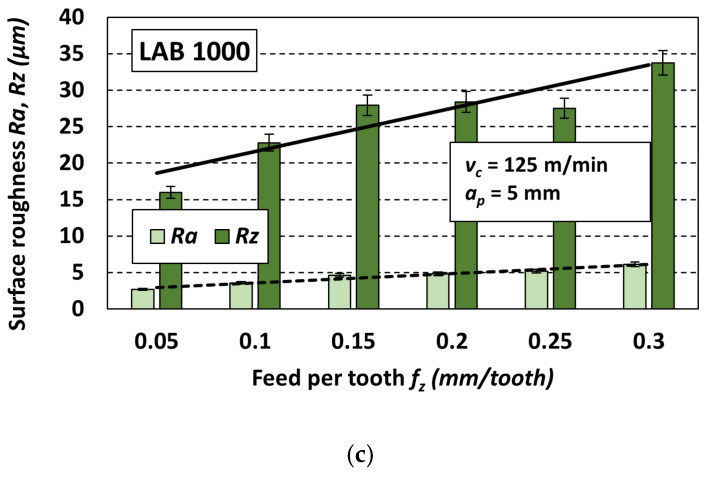
Example values of the roughness parameters *Ra* and *Rz* as a function of the feed per tooth *f_z_* for different PUB materials: (**a**) Labelite 45 PK, (**b**) Prolab 65 and (**c**) LAB 1000; values of constant cutting parameters *v_c_* = 125 m/min, *a_p_* = 5 mm.

Next, the third variable cutting parameter, the feed per tooth *f_z_*, was analyzed. Similar to the previously discussed cases, bar charts were created to better visualize the obtained surface roughness results. An example set of charts is shown in Figure 16. Each of the three charts corresponds to a different PUB material: Labelite 45 PK (Figure 16a), Prolab 65 (Figure 16b) and LAB 1000 (Figure 16c), milled with the same constant cutting parameters: *v_c_* = 125 m/min and *a_p_* = 5 mm, and six feed rates *f_z_*. It should be noted that the trend of changes in the *R* parameter values under the influence of changes in cutting speed for other sets of constant cutting parameters was similar.

As expected, for each of the three types of PUB materials, increasing the feed per tooth *f_z_* increases the values of both parameters describing surface roughness (*Ra* and *Rz*). This is consistent with the literature data, both in terms of the theoretical basis for the formation of roughness due to the replication of the cutting-edge geometry and the feed motion of the cutting tool itself [20], as well as experimental data, e.g., [37,38]. The significance tests on the effect of feed per tooth depending on the applied PUB material, using a Complete Random Design statistical program, showed that within the accepted range of variability, feed per tooth significantly affects the *Ra* and *Rz* parameter values during the machining of all tested materials (*F* ≥ *F_kr_*).

**Figure 17:**
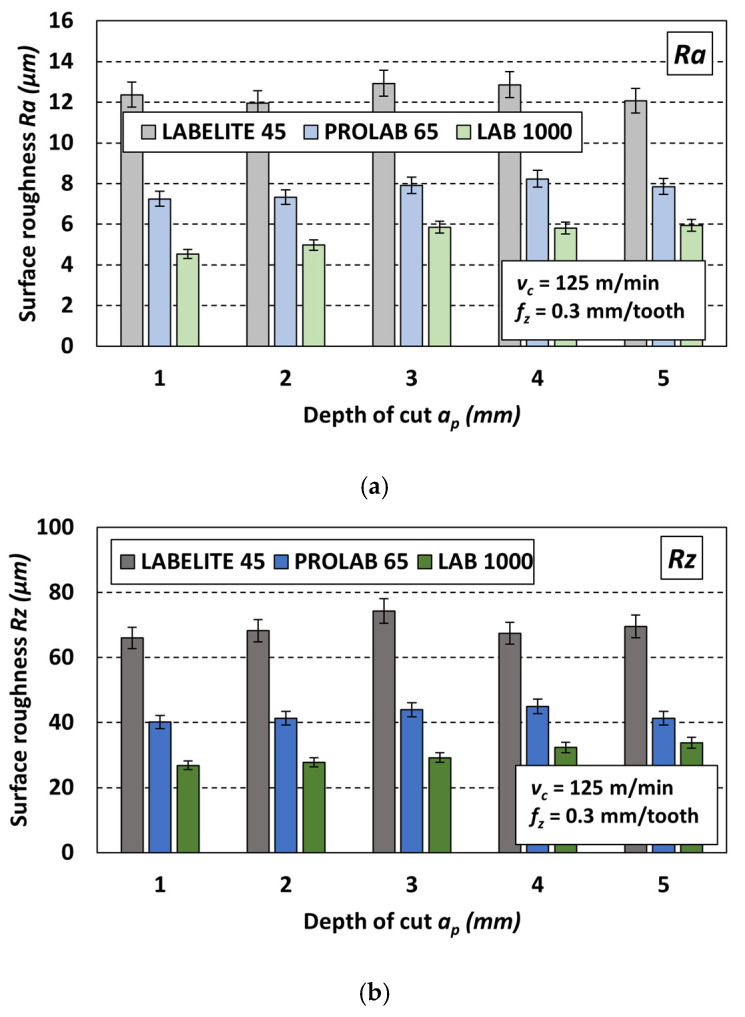
Influence of type of PUB material and variable feed per tooth *f_z_* on the surface roughness: (**a**) *Ra* and (**b**) *Rz*; values of constant cutting parameters *a_p_* = 5 mm, *v_c_* = 125 m/min.

Similarly to the previously described cases (variables: *v_c_* and *a_p_*), a significant effect of the density of the material from which the PUB is made on surface roughness was observed. Figure 17 shows example charts of changes in the *Ra* parameter (Figure 17a) and *Rz* parameter (Figure 17b) after milling with six feed rates *f_z_* and with constant cutting parameters: *v_c_* = 125 m/min and *a_p_* = 5 mm. It should be noted that the nature of changes in the *R* parameter values under the influence of changes in cutting speed for other sets of constant cutting parameters was similar.

As can be observed in Figure 17, similar to the previously discussed cases (variables: *v_c_* and *a_p_*), the highest roughness parameter value was obtained after milling the PUB with the lowest density (Labelite 45 PK), and the lowest for the surface of the PUB with the highest density (LAB 1000). The reason for this has already been explained earlier.

### 3.3. Tool Wear

**Figure 18:**
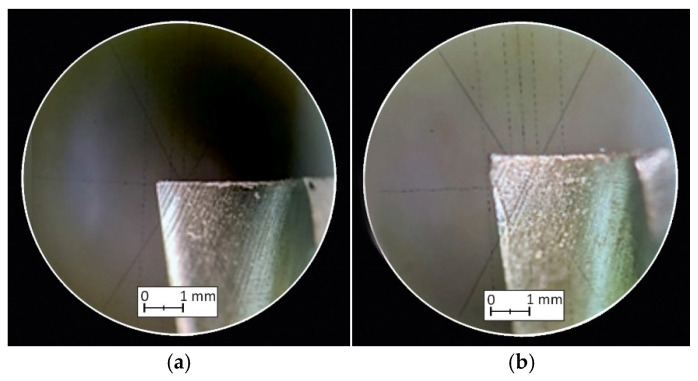

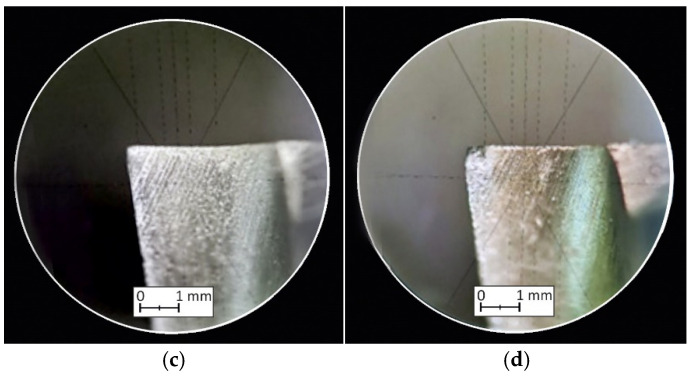
Outside edge wear: (**a**) fresh end mill, (**b**) after Labelite 45 PK machining, (**c**) after Prolab 65 machining and (**d**) after LAB 1000 machining.

**Figure 19:**
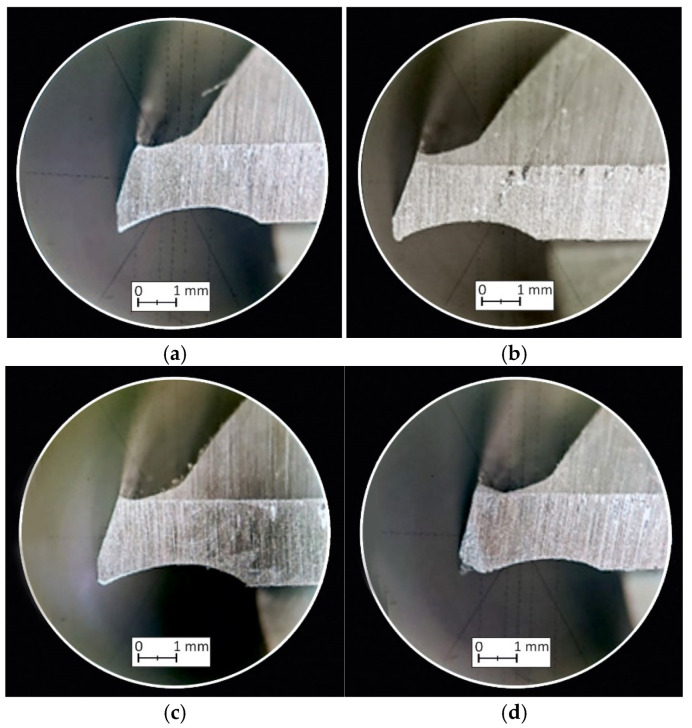
Flank wear: (**a**) fresh end mill, (**b**) after Labelite 45 PK machining, (**c**) after Prolab 65 machining and (**d**) after LAB 1000 machining.

**Figure 20:**
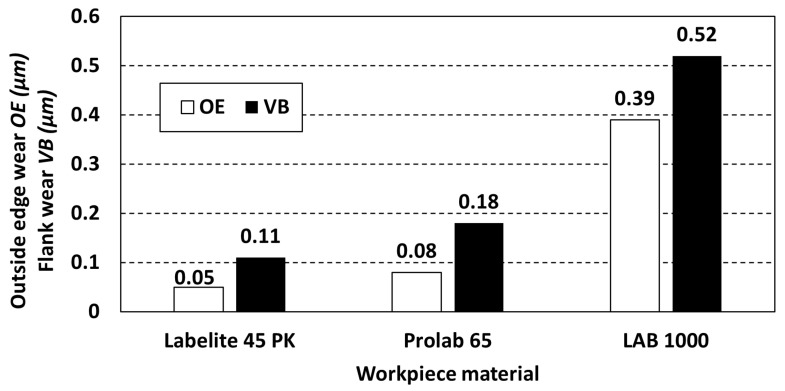
Wear indicators (*OE*, *VB*) for different workpiece materials.

Figure 18 and Figure 19 show microscopic images of the blades of the tested cutting tools taken to measure outside edge wear (*OE*) and flank wear (*VB*). Table 6 shows the results of the tool wear measurements determined by the *OE* and *VB* indicators, respectively, and a graphical illustration of the results is shown in Figure 20.

The analysis indicates that Labelite 45 PK (density: 0.45 g/cm^3^) results in the smallest tool wear, both in terms of *OE* and *VB*. As the density of the material increases, the wear rates increase as well. For Prolab 65 (density: 0.65 g/cm^3^), *OE* is 63% higher, while for LAB 1000 (density: 1.67 g/cm^3^), it is 680% higher than for PUB made from Labelite 45 PK. Similarly, the *VB* index measured on the blades is 61% greater than that measured for Labelite 45 PK after machining Prolab 65 and 373% greater after machining LAB 1000. It can therefore be concluded that tool wear increases with increasing material density, which results in higher cutting resistance during machining. Thus, tool wear for polyurethane materials increases with decreasing porosity of the material.

According to accepted standards, cutting edge wear should not exceed 0.2 mm for a 90° cutting edge angle or a maximum of 0.3 mm for a round-blade cutting tool [39]. Thus, for Labelite 45 PK and Prolab 65, the tool was still fit for use due to the low level of wear.

## 4. Summary and Conclusions

Three different types of polyurethane blocks were milled using a wide range of different cutting parameters. After machining, the roughness measurements *Ra* and *Rz*, which are responsible for the quality of the geometric structure of the surface, were carried out. This made it possible to assess how changes in PUB density, the cutting speed, feed rate and depth of cut affected the roughness of the machined surface. An analysis of the impact of the type of polyurethane material on tool wear was then carried out by measuring the cutting tool wear marks on the rake face and flank face after machining each of the tested materials. The tests carried out in this regard support the following conclusions:

- Taking into account surface quality, for all PUB materials, the following ranges of cutting parameters are recommended:
  ➢ cutting speed *v_c_* = 125 ÷ 175 m/min,
  ➢ feed per tooth *f_z_* = 0.05 ÷ 0.15 mm/tooth,
  ➢ depth of cut *a_p_* = 1 ÷ 5 mm.
- Cutting speed significantly affects the surface roughness after milling two PUB materials. For the material with the lowest density (Labelite 45 PK), increasing the cutting speed *v_c_* reduces the values of the roughness parameters *Ra* and *Rz*. The same trend was observed for the material with the highest density (LAB 1000). For the Prolab 65 material, cutting speed does not significantly affect the change in both *R* parameters.
- Feed rate significantly affects the values of surface roughness parameters after milling. Increasing the feed per tooth *f_z_* increases roughness parameters *Ra* and *Rz* regardless of PUB density.
- Changing the depth of cut *a_p_* has no noticeable effect on the resulting surface roughness of the workpiece, irrespective of its density.
- The best quality of the machined surface (low *Ra* and *Rz* values) was obtained for LAB 1000, followed by Prolab 65, and the worst for Labelite 45 PK. Thus, it can be found that the higher the material density, the lower the porosity and, consequently, the lower the surface roughness after milling.
- Tool wear described by *VB* and *OE* indicators increases along with the density of the machined polyurethane foam, successively for the material: Labelite 45K, Prolab 65 and LAB 1000. Thus, it can be concluded that a decrease in PUB porosity results in faster wear of the cutting tool blades.

In summary, it should be stated that the density of polyurethane foam has a significant impact on the surface roughness obtained after face milling PUB. As the density of the polyurethane foam increases, the overall level of the obtained roughness parameters *Ra* and *Rz* decreases, which translates into an improvement in the surface quality after machining. This fact should interest both those creating mock-up models or prototype models, as well as functional parts, e.g., used in control instruments. This knowledge can be utilized already at the stage of purchasing the appropriate type of polyurethane foam depending on individual needs.

The second important issue is the appropriate selection of cutting parameters, especially considering machining efficiency and production time. While changing the depth of cut *a_p_* does not significantly affect the *Ra* and *Rz* values, and changing the feed per tooth *f_z_* affects them in a predictable manner regardless of the material density, the effect of cutting speed *v_c_* on surface roughness should be considered individually in the context of PUB density. What is worth emphasizing from a practical point of view is the fact that using the highest cutting parameters ensures lower surface roughness compared to PUB materials.

From a practical point of view, the aspect of machining related to the cutting tool used is also important. In this case, increasing the density of PUB accelerates the wear of the cutting edge. Therefore, the tool should be carefully selected, considering its tool life in the context of production volume and associated time. The authors plan to investigate this topic in detail in their next work.

## Figures and Tables

**Table 1:** Physical and mechanical properties of polyurethane materials [25,26,27].

Properties	Unit	Labelite 45 PK	Prolab 65	LAB 1000
Density	g/cm^3^	0.45	0.65	1.67
Color	-	Pink	Beige	Gray
Flexural strength	MPa	12	34	90
Compressive strength	MPa	10	28	110
Thermal coefficient	10^−6^/K	55	75	50

**Table 2:** Technical data of EMCO Concept Mill 55 [28].

Properties	Unit	Value
Max. drive power	kW	0.75
Max. rotational speed	rpm	3500
Max. torque	Nm	3.7
Max. feed rate	m/min	2
Clamping area	mm	420 × 125
Number of tools	-	8
Machine weight	kg	220

**Table 3:** Technical data of an end mill [29].

Properties	Symbol	Unit	Value
Working diameter	*D*	mm	16
Shank diameter	*d*	mm	16
Working length	*l*	mm	19
Shank length	*l*_1_	mm	48
Length of a tool	*L*	mm	79
Flutes	*z*	-	2
Helix angle	*λ*	°	25

**Table 4:** Cutting parameters.

Parameter	Symbol	Unit	Value
Cutting speed	*v_c_*	m/min	25, 75, 125, 175
Feed per tooth	*f_z_*	mm/tooth	0.05, 0.1, 0.15, 0.2, 0.25, 0.3
Back engagement (depth of cut)	*a_p_*	mm	1, 2, 3, 4, 5
Working engagement (width of cut)	*a_e_*	mm	16

**Table 5:** Surface roughness measurement conditions [30].

Parameter	Symbol	Unit	Value
Stylus tip radius	*_rtip_*	μm	5
Diamond tip angle	-	°	90
Traverse speed	*_vt_*	mm/s	0.5
Measuring range	-	μm	±20
Tracing length	*LT*	mm	4.8
Evaluation length	*ln*	mm	4.0
Sampling length	*lr*	mm	0.8

**Table 6:** Comparison of outside edge wear (*OE*) and flank wear (*VB*).

Material	Labelite 45 PK	Prolab 65	LAB 1000
Outside edge wear *OE* (mm)	0.05	0.08	0.39
Flank wear *VB* (mm)	0.11	0.18	0.52

## Data Availability

Data are contained within the article.

## Nomenclature

*a_e_*	working engagement, width of cut (mm)
*a_p_*	back engagement, depth of cut (mm)
*f_z_*	feed per tooth (mm/tooth)
*OE*	outside edge wear (mm)
*N*	spindle rotational speed (rpm)
*Ra*	arithmetic average of the roughness profile height deviations from the mean line (µm)
*Rz*	average maximum height of the roughness profile (µm)
*VB*	flank wear (mm)
*v_c_*	cutting speed (m/min)
